# Pseudoaneurysm of an intercostal artery: endovascular treatment with PK papyrus coronary stent to prevent spinal ischemia

**DOI:** 10.1186/s42155-021-00211-z

**Published:** 2021-02-16

**Authors:** Julius Niehoff, Alexander Christian Bunck, David Maintz, Jan Robert Kroeger

**Affiliations:** 1 Department of Diagnostic and Interventional Radiology, Faculty of Medicine and University Hospital Cologne, University of Cologne, Kerpener Straße 62, 50937 Cologne, Germany; 2 Department of Radiology, Neuroradiology and Nuclear Medicine, Johannes Wesling University Hospital, Ruhr University Bochum, Bochum, Germany

**Keywords:** Endovascular treatment, Acute bleeding, Pseudoaneurysm, Stentgraft, Spinal ischemia

## Abstract

**Background:**

Endovascular treatment can be a fast and safe option in the case of acute, internal bleeding – but it requires special knowledge and technical skills. Interventionalists must consider the anatomy and potential complications. As in this case report, the anterior spinal artery, for example, can be a crucial vessel that must always be considered when embolizing intercostal or lumbar arteries. The risk of spinal ischemia has to be taken into account and should be minimized by choosing the appropriate treatment option.

**Case presentation:**

We report about a 77 year old, male patient with upper gastrointestinal bleeding after esophagectomy and gastric conduit reconstruction. A CT scan identified a pseudoaneurysm of an intercostal artery penetrating the gastric conduit as the bleeding source. In the DSA, a direct connection between the intercostal artery and the anterior spinal artery appeared to be likely. Due to the associated risk of spinal ischemia, an embolization of the intercostal artery was not an option. We decided to implant a stentgraft that would stop the perfusion of the pseudoaneurysm, but preserve the perfusion of the intercostal artery. Due to the small diameter of the vessel, we could not implant our commonly used stentgrafts in this case. Therefore, we chose an uncommon solution and used a stentgraft that is designed primarily for coronary arteries.

**Conclusions:**

Whenever intercostal or lumbar arteries need to be embolized, a possible connection to the anterior spinal artery must be considered and interventionalists have to be aware of possible ischemic complications. In this case, a stentgraft designed primarily for coronary arteries offered a good endovascular treatment option for the pseudoaneurysm of an intercostal artery. The risk of spinal ischemia could be minimized by using this stentgraft.

## Background

Over the past decades, endovascular treatment of acute, internal bleeding has become established in the clinical routine. Nowadays, interventionalists can choose from a great number of technical options. Among others, the individual anatomy and potential complications must be considered when choosing the most appropriate intervention.

Although manufacturers offer a wide variety of products, interventionalists continue to come across situations where off label use of a medical device is required. In fact, the off label use of medical products in the field of interventional radiology is widespread as clinical studies show (Zvavanjanja et al. 2012). Whenever interventionalists choose to use a product off label, it must be based on a sound medical opinion and must be in the best interest of the patient. Risks must always be considered carefully (Carrafiello et al. 2012).

We report about a case of acute bleeding originating from a pseudoaneurysm of an intercostal artery that required an unusual, off label solution because of potentially far reaching, ischemic complications.

## Case presentation

The 77 year old, male patient diagnosed with esophageal cancer underwent esophagectomy and gastric conduit reconstruction in an external hospital in January 2020. The patient suffered from an insufficiency of the intrathoracic anastomosis of the gastric conduit, which was treated by implanting esophageal stents.

**Fig. 1:**
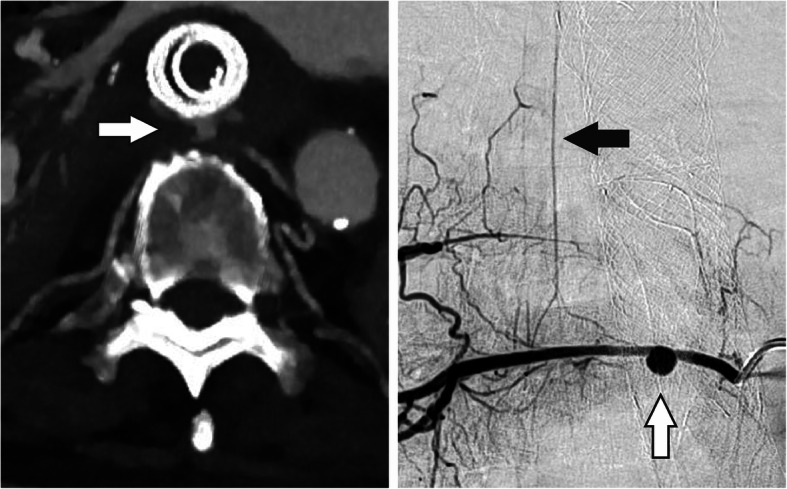
DSA and CT scan before implantation of the *PK Papyrus Stent. *In the CT scan the pseudoaneurysm can be detected ventral of the spine with direct contact to the esophagus. Furthermore, the previously implanted, esophageal stents can be seen. The esophageal tissue seems to be thinned out. After the CT scan, the DSA confirms the suspected pseudoaneurysm and shows the connection between the intercostal artery and the anterior spinal artery. White arrow: Pseudoaneurysm of the intercostal artery. Black arrow: Anterior spinal artery

After an episode of upper gastrointestinal (GI) bleeding that led to a hemorrhagic shock, the patient was transferred to the tertiary care hospital. Over the next few days, the patient presented further episodes of upper GI bleeding, which could not be controlled by endoscopy. In a CT scan we identified a pseudoaneurysm of an intercostal artery dorsal of the gastric conduit (see Fig. 1). After an interdisciplinary discussion, we decided in favor of an endovascular therapy for the pseudoaneurysm.

The angiography was performed under general anesthesia. Access was gained via a retrograde 6 French sheath in the right common femoral artery. The intercostal artery of the right, eighth intercostal space was probed with a 6 French Guider Softip XF Catheter (Boston Scientific). Digital subtraction angiographies (DSA) were made from different projections. The maximum diameter of the pseudoaneurysm was 8 mm (see Fig. 1).

The DSA images also showed a fine, vertical vessel projected onto the spine, highly suspicious of being the anterior spinal artery. The direct connection between that vessel and the intercostal artery could not be identified clearly. Also we were not able to evaluate with certainty whether this vessel would be collateralized from the opposite side if we embolized the intercostal artery completely.

Consequently, we decided to implant a stentgraft into the intercostal artery that covered the pseudoaneurysm but maintained the blood flow within the artery and all uncovered side branches of the artery. Because of the small diameter of the intercostal artery (3 mm), we chose the *PK Papyrus Coronary Stent* (*Biotronik*, 3 mm x 15 mm).

**Fig. 2:**
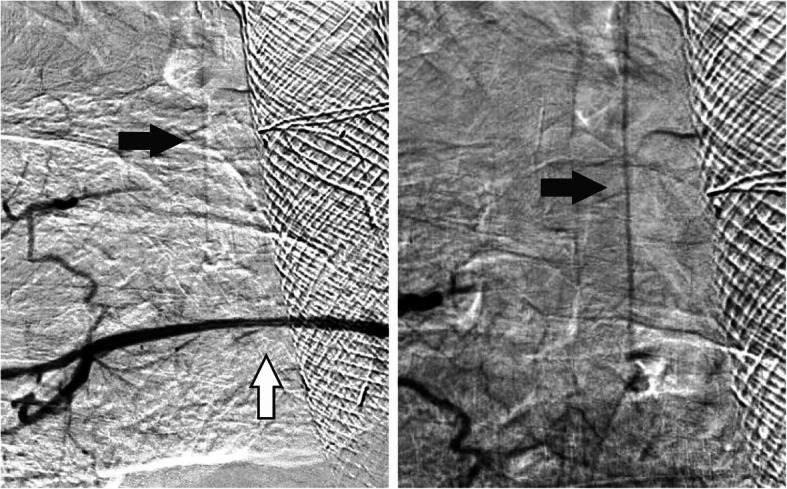
DSA after implantation of the *PK Papyrus Stent. *After implanting the stentgraft in the proximal segment of the intercostal artery, the pseudoaneurysm is no longer perfused (white arrow) minimizing the risk of recurrent bleeding. At the same time, the perfusion of the anterior spinal artery is preserved (black arrow) minimizing the risk of spinal ischemia

First, we had to find a stable position in the ostium of the intercostal artery using the 6 French Guider Softip XF Catheter. Then, the intercostal artery was probed with a 0.014” guide wire. Finally, the balloon expandable stent was placed safely and precisely in the intercostal artery to cover the pseudoaneurysm. In the final DSA, the pseudoaneurysm could no longer be delineated while the perfusion of the anterior spinal artery was preserved (see Fig. 2).

The postinterventional clinical course in the tertiary care hospital was uneventful without further episodes of upper GI bleeding. Unfortunately, during the preparation of this case report, we received the news that the patient had passed away in another hospital a few weeks after the intervention.

## Discussion

The contribution of intercostal and lumbar arteries to the perfusion of the anterior spinal artery has been well described in the literature (Uezu et al. 2003). Spinal ischemia with paraplegia is a serious complication that can occur during different surgeries and endovascular interventions, respectively.

Brown et al. reported about a case of anterior spinal cord infarction following bronchial artery embolization - although none of the embolized vessels showed anterior spinal artery feeders in the DSA (Brown and Ray 2012). Likewise, Gaudry et al. described an accidental migration of a liquid embolic agent into the anterior spinal artery during lumbar artery embolization in an animal model (Gaudry et al. 2018). Matsuda et al. report about an incidence of up to 12.5 % of permanent and transient paraplegia following thoracic endovascular aneurysm repair (TEVAR) due to spinal cord injury (Matsuda et al. 2010).

In our case, the use of vascular coils or liquid embolic agents would have been associated with a risk of spinal ischemia. Therefore, we decided to use a stentgraft in this case. The anatomy of our patient also favored the use of a stentgraft because the affected intercostal artery had a straight, horizontal vascular course in the segment where the pseudoaneurysm was located and there were no other vessels originating from this section of the intercostal artery.

Since the maximum diameter of the intercostal artery was only 3 mm, we used a stentgraft primarily designed for coronary arteries. The *PK Papyrus Stent* is a covered, cobalt chromium, non-woven, electrospun polyurethane stent that is actually designed to seal coronary artery perforations. The stent is balloon expandable and can be delivered via a 0.014” guide wire. The characteristics of this stent – smallest available diameter is 2.5 mm, low crossing profile and high flexibility – made the *PK Papyrus Stent* the best solution in our case.

## Conclusions

The anterior spinal artery and the possible ischemic complications must be considered when intercostal or lumbar arteries are embolized – especially when the proximal segment of the arteries is affected. This applies to a scenario in which the anterior spinal artery contrasts in DSA as well as to a scenario in which the artery cannot be identified in DSA. Due to the small diameter, stentgrafts designed primarily for coronary arteries can offer a good endovascular treatment option in the case of acute bleeding or pseudoaneurysms of small caliber vessels.

## Data Availability

Data sharing is not applicable to this article as no datasets were generated or analyzed during the current study.
